# Pediatric oncology clinician communication about sexual health with adolescents and young adults: A report from the children’s oncology group

**DOI:** 10.1002/cam4.4077

**Published:** 2021-06-15

**Authors:** Natasha N. Frederick, Kristin Bingen, Sharon L. Bober, Brooke Cherven, Xinxin Xu, Gwendolyn P. Quinn, Lingyun Ji, David R. Freyer

**Affiliations:** ^1^ Center for Cancer and Blood Disorders Connecticut Children’s Medical Center Hartford CT USA; ^2^ University of Connecticut College of Medicine Storrs CT USA; ^3^ Department of Pediatrics Medical College of Wisconsin Milwaukee WI USA; ^4^ Department of Psychosocial Oncology and Palliative Care Dana‐Farber Cancer Institute Boston MA USA; ^5^ Aflac Cancer and Blood Disorders Center at Children’s Healthcare of Atlanta Atlanta GA USA; ^6^ Emory University School of Medicine Atlanta GA USA; ^7^ Children’s Oncology Group Monrovia CA USA; ^8^ Departments of OB‐GYN Population Health Division of Medical Ethics Grossman School of Medicine New York University New York NY USA; ^9^ Department of Preventive Medicine Keck School of Medicine University of Southern California Los Angeles CA USA; ^10^ Cancer and Blood Disease Institute Children’s Hospital Los Angeles USC Norris Comprehensive Cancer Center Los Angeles CA USA; ^11^ Keck School of Medicine University of Southern California Los Angeles CA USA

**Keywords:** adolescent and young adult, sexual and reproductive health in cancer, sexual health education, sexual health in pediatric oncology

## Abstract

**Background:**

Sexual health (SH) is an important concern for adolescents and young adults (AYAs). This study determined current SH communication practices, barriers, and additional resources needed among pediatric oncology clinicians who treat AYAs.

**Methods:**

A cross‐sectional survey was developed by the Children's Oncology Group (COG) AYA Committee and sent to pediatric oncologists (n = 1,987; 85.9%) and advanced practice providers (APPs, n = 326; 14.1%) at 226 COG institutions. Responses were tabulated and compared using tests of proportion and trend.

**Results:**

The sample comprised 602 respondents from 168 institutions and was proportionally representative (468 oncologists [77.7%], 76 APPs [12.6%], 58 unidentified [9.6%]; institutional and provider response rates 74.3% and 26.2%, respectively). Almost half of respondents (41.7%) reported no/small role in SH care. Medical topics were discussed most often, including contraception (67.2%), puberty (43.5%), and sexual activity (37.5%). Topics never/rarely discussed included gender identity (64.5%), sexual orientation (53.7%), and sexual function (50.3%). Frequently cited communication barriers included lack of time, low priority, perceived patient discomfort, and the presence of a parent/guardian. Respondents endorsed the need for further education/resources on sexual function (66.1%), gender identity/sexual orientation (59.5%), and body image (46.6%). Preferred education modalities included dissemination of published guidelines (64.7%), skills training modules (62.9%), and webinars (45.3%). By provider type, responses were similar overall but differed for perception of role, barriers identified, and resources desired.

**Conclusions:**

Many pediatric oncology clinicians play minimal roles in SH care of AYAs and most SH topics are rarely discussed. Provider‐directed education/training interventions have potential for improving SH care of AYA cancer patients.

## Graphical abstract

In this cross‐sectional survey, pediatric oncology clinicians recognized the importance of communicating with adolescents and young adults about sexual health (SH) but reported multiple barriers related to lack of content expertise and experience. Participants identified educational resources that would enhance SH communication, including published guidelines, training curricula/modules, and webinars.

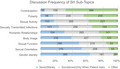

## 1 INTRODUCTION

Exploring sexuality while navigating physical, social, and emotional changes is a normative developmental task of adolescence and young adulthood. This holds true for adolescents and young adults (AYAs) with cancer, who are at increased risk for sexual health (SH) concerns and adverse sexual and reproductive health outcomes during treatment and survivorship. As part of comprehensive cancer care, these vulnerable patients require sexual and reproductive health education that is medically accurate and developmentally appropriate, along with access to relevant clinical services.^1^

Participation in risky SH behaviors, such as using unreliable or inconsistent contraception, is common among adolescents but may carry more serious consequences for AYAs with cancer. Research demonstrates AYA patients and survivors engage in unsafe sexual behaviors at similar or higher rates than healthy peers.^2^, ^3^, ^4^, ^5^ Additionally, AYA survivors are more likely to experience sexual dysfunction and decreased libido, which negatively impact intimate relationships, sexual satisfaction, and self‐esteem.^6^, ^7^, ^8^, ^9^ Furthermore, AYAs have concerns that influence romantic relationships and body image, such as social disclosure of cancer history, feeling different from peers, and potential infertility.^8^

Despite these needs, little is known about communication between oncologists and AYAs concerning SH beyond fertility, including counseling on safe sex practices and contraception during therapy, and assessing sexual dysfunction.^3^, ^10^, ^11^, ^12^ In part, communication deficits in SH stem from poor clinician insight into psychosexual issues experienced by AYAs who, in turn, express desire for such conversations.^11^, ^13^, ^14^, ^15^, ^16^ A qualitative study shows key barriers to clinician‐led SH communication include lack of education/training, knowledge and communication skills, low awareness of specific issues faced by AYAs during and following cancer treatment, and lack of competency in managing identified problems.^17^

Currently, SH communication practices between pediatric oncology clinicians and AYAs are not well described. To address this gap on a national scale, we surveyed pediatric oncologists and advanced practice providers (APPs) in the Children's Oncology Group (COG) to determine clinician perspectives on SH communication practices, barriers, the need for additional education/training and resources, and preferred modalities for their delivery. The overall goal was to identify opportunities for developing provider‐directed interventions to improve SH communication between pediatric oncology clinicians and AYAs treated at COG institutions.

## 2 METHODS

This multicenter, cross‐sectional survey was deemed exempt by the Institutional Review Board at Connecticut Children's.

### 2.1 Participants

Study participants were pediatric oncologists and APPs who provide care for AYA oncology patients (ages 15–29 years old) and are members of COG. Surveys were sent to pediatric oncologists (n = 1,987; 85.9%) and advanced practice providers (APPs, n = 326; 14.1%) at 226 COG institutions identified through COG clinician listservs. Anticipating potential challenges in clinician recruitment to a survey study, we aimed to achieve more than 60% representation of COG institutions.^18^ Non‐participants were those who were invited but did not respond; consequently, no descriptive information is available for this group.

### 2.2 Sexual health survey

Members of the Sexual Health Task Force of the COG AYA Oncology Discipline Committee, including pediatric oncology physicians, APPs, nurses, and psychologists, developed the survey content. The survey included 18 questions regarding individual attitudes and current practices in SH communication (4), barriers to SH communication (1), optimal education and resource needs and preferred modalities of delivery (4), perceived value of SH discussions with AYAs (2), and demographics (7) (Appendix 1). Question format included multiple choice, Likert scale, and rank order.

Prior to administration, the COG AYA, Nursing, Cancer Control and Supportive Care, and Outcomes and Survivorship Committees reviewed the survey. The survey underwent pilot testing with five clinicians to determine comprehension, acceptability, and estimated time for completion and thus feedback resulted in minimal changes. The survey took 5–10 min to complete.

The survey was administered via the Research Electronic Data Capture (REDCap) platform. Personalized links were emailed to potential participants, with follow‐up emails to non‐responders at 2, 4, and 6 weeks as per the Dillman Method.^19^ The survey remained open for 6 months between March and September, 2019. Participant personal information (name and email address) was confidential and was not associated with survey responses.

### 2.3 Data analyses

Survey responses were tabulated and compared between physicians and APPs using Pearson's Chi‐squared test, Fisher's exact test, or test of trend as appropriate. All analyses were performed using the Statistical Analysis System (SAS) statistical software package, version 9.4. All reported *p* values are two‐sided and ≤0.05 was considered to be statistically significant.

## 3 RESULTS

### 3.1 Participant characteristics

The sample comprised 602 respondents (468 oncologists [77.7%], 76 APPs [12.6%], and 58 unidentified [9.6%]), representing 168 institutions (provider and institutional response rates were 26.2% and 74.3%, respectively) (Table 1). Participants with known provider type reflected the overall COG cohort with 86.0% physicians and 14.0% APPs. Most were female (67.0%) and had 16–20 years of pediatric oncology experience (56.5%). Compared with APPs, physicians were more likely to report male gender (*p *< 0.001). Compared with physicians, APPs reported caring for more AYAs patients between 15 and 29 years old (*p *= 0.003), working in a setting with upper age limit ≥25 years for pediatrics (*p *= 0.003), and having formal training in AYA SH issues (*p *= 0.033). Most respondents completed all questions (544, 90.4%). Comparison of clinician responses based on self‐identified gender revealed no significant differences regarding barriers to SH communication on specific SH sub‐topics or role in discussing SH.

**TABLE 1:** Participant characteristics (n = 602)

Variables	All participants
	n (%)
Clinician type	
Physician	468 (85.9)
Advanced practice provider	76 (13.9)
Unknown^a^	58
Gender	
Female	364 (67.0)
Male	178 (32.8)
Other	1 (0.2)
Decline^a^	2
Unknown^a^	57
Years working in pediatric oncology	
1–5	96 (17.6)
6–10	137 (25.2)
11–20	170 (31.3)
21–30	103 (18.9)
31–40	36 (6.6)
>40	2 (0.4)
Unknown^a^	58
% of patients between ages 15 and 29 years	
0–20	166 (30.5)
21–40	259 (47.5)
41–60	84 (15.4)
61–80	28 (5.1)
81–100	8 (1.5)
Unknown^a^	57
Number of new oncology patients per year	
<50	103 (18.9)
51–100	156 (28.7)
101–150	90 (16.5)
>150	195 (35.8)
Unknown^a^	58
Maximum age range for new patients to receive cancer therapy within your division	
15 years	9 (1.7)
18 years	77 (14.2)
21 years	176 (32.4)
25 years	145 (26.7)
30 years	72 (13.2)
35 years	20 (3.7)
≥40 years	17 (3.1)
No upper age limit	28 (5.1)
Unknown^a^	58
Prior formal training in addressing sexual health issues with AYAs	
Yes	62 (11.4)
No	482 (88.6)
Unknown^a^	58

^a^ The unknown/decline categories were excluded from the calculation of percentages.

### 3.2 Clinician communication practices

**FIGURE 1:**
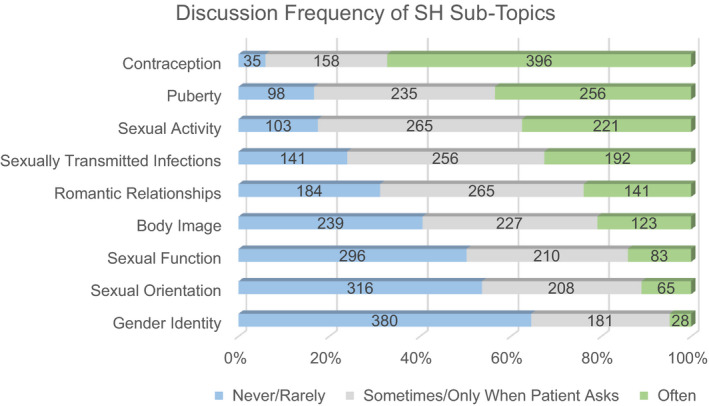
Frequency of clinician conversations with AYAs on specific sexual health topics. These data include physicians and APPs combined

Of 599 participants with known provider type, 251 (41.9%) reported having no/small role in SH care (Table 2). Approximately 80% of both oncologists and APPs combined reported that either an oncologist or APP, as opposed to other team members, should take primary responsibility for addressing SH with AYAs. Nearly a third (31.4%) waited until patients were between 16 and 18 years old to start SH conversations. Figure 1 illustrates frequency of SH discussions by topic.

**TABLE 2:** Current sexual health communication practices

	All participants n = 602	Physician n = 468	APP n = 76	*p* value
	n(%)	n(%)	n(%)	
How much of a role do you play in the discussion of sexual health care with your AYA patients?				0.96^b^
No role	20 (3.3)	13 (2.8)	5 (6.6)	
Small role	231 (38.6)	180 (38.5)	27 (35.5)	
Moderate	199 (33.2)	158 (33.8)	21 (27.6)	
Major/very major role	149 (24.9)	117 (25.0)	23 (30.3)	
Unknown^a^	3	0	0	
Who should take primary responsibility for discussing sexual health with AYA patients?				<0.001^c^
Oncologist	380 (63.5)	306 (65.4)	35 (46.1)	
Oncology APP	112 (18.7)	68 (14.5)	33 (43.4)	
Social work	10 (1.7)	10 (2.1)	0	
Oncology nurse	14 (2.3)	11 (2.4)	1 (1.3)	
Endocrinologist	9 (1.5)	5 (1.1)	3 (3.9)	
Psychologist	28 (4.7)	27 (5.8)	1 (1.3)	
Child life specialist	1 (0.2)	1 (0.2)	0	
Patient navigator	3 (0.5)	3 (0.6)	0	
Other	41 (6.9)	37 (7.9)	3 (3.9)	
Unknown^a^	4	0	0	
At what age do you tend to start discussing sexual health with your AYA patients?				0.022^c^
Not discussed	38 (6.4)	33 (7.1)	3 (3.9)	
Before age 13 years	58 (9.7)	43 (9.2)	10 (13.2)	
Age 13–15 years	296 (49.5)	219 (46.8)	48 (63.2)	
Age 16–18 years	188 (31.4)	157 (33.5)	14 (18.4)	
After age 18 years	18 (3.0)	16 (3.4)	1 (1.3)	
Unknown^a^	4	0	0	

^a^ The unknown/decline categories were excluded from the calculation of percentages.

^b^ Test of trend.

^c^ Fisher's exact test.

### 3.3 Communication barriers

Prevalent barriers reported across all SH topics included lack of time, perceived patient discomfort, and presence of a parent/guardian (Table 3). When compared with physicians, APPs were more likely to report communication barriers about sexually transmitted infections (STIs) (60.5% vs. 48.1%, respectively; *p *= 0.044) but less likely for body image (50.0% vs. 64.7%, respectively; *p *= 0.014).

**TABLE 3:** Communication barriers reported for each sexual health topic

Barriers	All participants n(%)								
	N	Lack of knowledge	Lack of resources	Low priority	My personal discomfort	Lack of time	Patient discomfort	Parent or guardian present	No barrier
Puberty	551	20 (3.6)	27 (4.9)	114 (20.7)	17 (3.1)	149 (27.0) (27.0)	88 (16.0)	55 (10.0)	272 (49.4)
Sexual activity	550	20 (3.6)	33 (6.0)	74 (13.5)	72 (13.1)	151 (27.5) (27.5)	191 (34.7)	234 (42.5)	165 (30.0)
Sexual orientation	549	74 (13.5)	63 (11.5)	155 (28.2)	92 (16.8)	156 (28.4) (28.4)	145 (26.4)	157 (28.6)	112 (20.4)
Gender identity	553	144 (26.0)	90 (16.3)	158 (28.6)	100 (18.1)	131 (23.7)	107 (19.3)	136 (24.6)	95 (17.2)
Sexually transmitted infections	553	12 (2.2)	22 (4.0)	62 (11.2)	20 (3.6)	109 (19.7)	70 (12.7)	129 (23.3)	283 (51.2)
Contraception	553	21 (3.8)	23 (4.2)	32 (5.8)	8 (1.4)	83 (15.0)	49 (8.9)	105 (19.0)	354 (64.0)
Body image	553	80 (14.5)	74 (13.4)	117 (21.2)	23 (4.2)	150 (27.1)	60 (10.8)	30 (5.4)	212 (38.3)
Sexual dysfunction	553	145 (26.2)	76 (13.7)	120 (21.7)	79 (14.3)	123 (22.2)	143 (25.9)	102 (18.4)	131 (23.7)
Romantic relationships	553	18 (3.3)	22 (4.0)	130 (23.5)	18 (3.3)	145 (26.2)	68 (12.3)	73 (13.2)	232 (42.0)

Note:

Participants were able to choose more than one barrier for each topic

### 3.4 Education and resource needs

Participants reported on education/resource needs and preferred modalities for acquiring information. (Table 4) Whereas approximately one third of providers cited barriers to addressing contraception and approximately half reported barriers to addressing puberty and STIs, more than 75% of respondents reported barriers to discussing gender identity, sexual orientation, and sexual function. APPs were more likely to report the need for further education on STIs (35.5% vs. 15.4%; *p *< 0.001) and how to take an SH history (40.0% vs. 20.4%; *p *< 0.001), whereas physicians were more likely to report the need for further education on body image (49.1% vs. 31.6%; *p *= 0.004) and how to build a local network of SH specialists (47.0% vs. 34.7%; *p *= 0.047). Respondents expressed a strong desire for improved online information and resources (Table 5).

**TABLE 4:** Clinician‐reported education needs (n = 602)

	All participants
	n(%)
Top sexual health topics for which participants require further education:	
Sexual function	364 (66.1)
Gender identity/sexual orientation	328 (59.5)
Body image	257 (46.6)
Sexual activity	190 (34.5)
Contraception	173 (31.4)
Puberty	107 (19.4)
Sexually transmitted infections	99 (18.0)
Romantic relationships	72 (13.1)
Other	38(6.9)
Unknown^a^	51
Skill sets in which further education would be most helpful:	
Impact of cancer and cancer therapy on sexual health	350 (64.7)
How to talk to AYAs about sexual health issues	310 (57.3)
How to identify problems with sexual function	292 (54.0)
How to treat common sexual health problems	268 (49.5)
How to build a local network of sexual health specialists to assist with patient care	244 (45.1)
How to take a sexual health history	124 (22.9)
Other	11 (2.0)
Unknown^a^	61
Preferred education modality:	
COG or national guidelines	347 (64.7)
Clinician training curricula or modules	337 (62.9)
ASPHO/APHON webinar	243 (45.3)
Age‐appropriate patient‐reported outcome measures to assess patient sexual health care needs	242 (45.1)
Standardized partnership with sexual health specialists	204 (38.1)
Sessions/small groups/workshops at national professional meetings	181 (33.8)
Other	10 (1.9)
Unknown^a^	66

Abbreviations: APHON, Association of Pediatric Hematology/Oncology Nurses; ASPHO, American Society of Pediatric Hematology/Oncology; COG, Children's Oncology Group.

^a^ The unknown/decline categories were excluded from the calculation of percentages.

**TABLE 5:** Clinician‐identified resource needs (n = 602)

Resource needs	All participants
	n(%)
Written pamphlets or booklets	
Very helpful or extremely helpful	263 (48.1)
Somewhat helpful	162 (29.6)
Not at all helpful or slightly helpful	122 (22.3)
Unknown^a^	55
Online information and resources	
Very helpful or extremely helpful	414 (75.8)
Somewhat helpful	110 (20.1)
Not at all helpful or slightly helpful	22 (4.0)
Unknown^a^	56
Video‐based education modules	
Very helpful or extremely helpful	261 (47.7)
Somewhat helpful	186 (34.0)
Not at all helpful or slightly helpful	100 (18.3)
Unknown^a^	55

^a^ The unknown/decline categories were excluded from the calculation of percentages.

## 4 DISCUSSION

This study represents the first large‐scale survey of pediatric oncology clinicians on perceived SH communication practices with the goal of identifying education/resource needs to improve SH conversations. While many participants reported playing a minimal role in SH communication with AYAs, the majority identified themselves as the clinician who should be responsible for ensuring these conversations take place. When such discussions do occur, clinicians are more apt to focus on the medical aspects of SH, such as contraception, puberty, and STI risk, rather than sexual function, sexual orientation, and gender identity. Participants expressed a need for further education and guidelines on screening, management, and communication about SH in AYA patients. These findings highlight the existing gaps in clinical knowledge and practice, thereby informing future clinician‐centered education strategies and areas needing further research. They also serve as a contemporary initial benchmark for future comparison.

This study indicates the discussions of SH with AYAs during and post‐cancer treatment do not occur with the frequency and depth recommended by the American Academy of Pediatrics (AAP), the National Comprehensive Cancer Network (NCCN), and the American Society of Clinical Oncology (ASCO)^17^, ^20^, ^21^, ^22^, ^23^ Our results align with previous studies examining AYAs recall of SH discussions, showing low frequency.^22^, ^24^ When SH is addressed in the oncology setting, it is often entangled in or simply subsumed by discussion of fertility risk.^25^ In contrast, this study carefully delineated individual SH concepts.

Nearly 42% of participants acknowledged having little to no role in discussing SH with AYAs. These concerning findings suggest many AYAs are not given the opportunity to address key SH concerns, as prior research makes clear AYAs are highly unlikely to initiate conversations regarding SH.^24^ Interestingly, physicians and APPs each demonstrated strong preferences for ownership of these conversations as opposed to assigning the responsibility to social work or psychology, which highlights an encouraging desire to address SH as part of comprehensive cancer care and presents an opportunity for more communication training.

About one third of participants reported not initiating SH conversations with patients less than 16–18 years old, which likely results in missed opportunities for patient education, as the average age of sexual debut in the United States is 16 years.^26^ Nearly half (46.8%) of all high school students have a history of sexual intercourse, with only 59.1% reporting condom use during their last sexual encounter.^26^ These data underscore the need for educating clinicians about AYA sex practices and introducing such conversations in a developmentally appropriate manner. Clinicians may also benefit from learning how to partner with psychology, social work, and other supportive care colleagues in providing appropriate SH care.

AYAs with cancer face increased risk for sexual dysfunction during and after treatment.^6^, ^7^, ^8^, ^9^ Several studies indicate that over 30% of childhood cancer survivors go on to experience sexual dysfunction.^27^, ^28^, ^29^ Compared with older adults, SH problems are often more severe and distressing in AYAs as many childhood cancer survivors experience delays in dating, marriage, and sexual debut.^3^, ^30^, ^31^, ^32^, ^33^ When cancer is diagnosed during adolescence, survivors are more likely to experience impaired sexual function and decreased libido compared with those diagnosed early in childhood.^6^, ^7^, ^8^ Despite this, sexual function was reported as never/rarely discussed by half of the clinicians in this study. This is particularly concerning because dysfunction tends not to improve over time, therefore, patients may suffer these complications indefinitely if not addressed.^34^

Clinician‐reported barriers to SH communication identified here are similar to those reported in prior qualitative research in AYAs, fertility‐focused communication studies, and studies exploring subspecialty pediatric clinician communication in cystic fibrosis and perinatally diagnosed HIV.^24^, ^35^, ^36^, ^37^ Research in adult cancer populations illustrates similar barriers to patient–clinician SH communication as reported by medical oncologists.^38^ Based on these findings, interventions developed to improve pediatric oncology clinician‐AYA SH communication may also benefit medical oncologists caring for AYAs as well as clinicians of non‐cancer AYA population. In this study, over 40% of participants identified the presence of a parent/family member as a barrier to conversations about sexual activity, highlighting the need for clinicians to speak to AYAs alone. While communication barriers reported by physicians and APPs were consistent across most SH topics, there were some differences, suggestive of variances in training background. When compared with physicians, APPs were more likely to report communication barriers about STIs but less likely about body image. These findings are important when considering potential provider‐directed education interventions.

While “lack of knowledge” was not often identified as a barrier to communication on specific SH topics with AYAs, more than 50% of participants expressed the need for further education in the areas of sexual function and gender identity/sexual orientation, and more than 30% reported a need for more education on body image, sexual activity/safe sex practices, and contraception. Based on these findings, we anticipate further clinician education will help to improve clinician knowledge, comfort, and communication on SH topics relevant to the AYA patient. Education efforts may also help to address the barrier of “low priority” by highlighting the significance SH has for an AYA patient's overall well‐being. Improving clinician knowledge of gender identities and sexual orientation and how SH needs may differ is an important step in ensuring all conversations are inclusive and not heteronormative. These findings suggest many clinicians are aware of some SH topics but are uncomfortable or ill‐prepared for adequate discussion about other pertinent aspects of SH.^17^ This is not surprising given that most physician knowledge about SH is acquired during general pediatrics residency with minimal structured education on SH during pediatric hematology/oncology fellowship training.^39^, ^40^ Future intervention strategies should emphasize AYA‐reported communication needs and the importance of clinicians initiating conversations.^24^ Participants identified published guidelines and online education modules as the preferred modalities for supplemental SH education. While published screening recommendations exist for sexual dysfunction screening and SH communication with AYA cancer patients and survivors, they are limited in scope and detail, thereby creating opportunities for improvement.^20^, ^21^, ^41^

Participants also identified online modules as a preferred modality for SH education. Benefits of this approach include accessibility, efficacy, cost effectiveness, and learner flexibility and interactivity; however, such strategies must be carefully designed to focus on improvement in clinician practice and patient outcomes.^42^ This strategy has worked successfully in the setting of oncofertility education for nursing and other allied health professionals, as well as for oncologists regarding gender minority health care needs.^43^, ^44^

This study has several strengths and some limitations. Significant strengths include a relatively large, proportionally representative sample of approximately 600 clinicians from 168 COG institutions, inclusion of both pediatric oncologists and APPs, a contemporary survey that carefully delineated multiple SH topics other than fertility, and solicitation of preferences that can assist in the development of provider‐directed educational interventions. Although our participant response rate was lower than desired in this non‐incentivized survey, it falls within the range of physician response rates observed in previous survey studies (12%–50%).^18^ Our results could represent a “best case scenario” because non‐participants may have had less interest in, and felt even less prepared, to address AYA SH than did participants. We were unable to fully assess the representativeness of our sample because, to protect confidentiality, we could not collect demographic data on non‐participants. Nevertheless, the large absolute number of participants, coupled with a high institutional response rate and distribution of provider type that reflects the pool of invited COG clinicians, our results are likely valid and generalizable. Finally, this survey does not capture the quality of the current SH conversations taking place between clinicians and AYAs. Prior qualitative research supports there is room for improvement and further research is warranted.

This study emphasizes a need for further clinician education in SH communication with AYA patients, which includes the development of more detailed practice guidelines that could be disseminated through COG and the creation of educational strategies, namely through e‐learning modalities, that will expand access for the busy clinician. Screening strategies should be tailored to the AYA patient to identify those most at risk for SH problems, which may then be tested in the cooperative group research setting. Additional analyses exploring the individual characteristics of clinicians more likely to report SH communication with AYAs may be helpful in intervention development.

## 5 ETHICAL APPROVAL STATEMENT

This study was deemed exempt by the Institutional Review Board at Connecticut Children's (IRB # 18–154) prior to commencing this study.

## 6 ETHICS STATEMENT

This study was deemed exempt by the Institutional Review Board at Connecticut Children's.

## CONFLICT OF INTEREST

The authors have no conflict of interest.

## AUTHOR CONTRIBUTION

**Natasha Frederick**: conceptualization, data curation, formal analysis, methodology, project administration, writing‐original draft, writing‐review and editing. **Kristin Bingen:** conceptualization, formal analysis, methodology, writing‐review and editing. **Sharon Bober:** conceptualization, formal analysis, methodology, writing‐review and editing. **Brooke Cherven:** conceptualization, formal analysis, methodology, writing‐review and editing. **XinXin Xu:** formal analysis, writing‐review and editing. **Gwendolyn Quinn:** conceptualization, formal analysis, methodology, writing‐review and editing. **Lingyun Ji:** formal analysis, writing‐review and editing. **David R. Freyer:** conceptualization, data curation, methodology, formal analysis, writing‐review and editing.

## Data Availability

The data that support the findings of this study are available from the corresponding author upon reasonable request.
